# A unique case of well-differentiated gastric-type adenocarcinoma coexisting with a gastric adenocarcinoma of the fundic gland in a Helicobacter pylori-uninfected stomach

**DOI:** 10.1055/a-2173-7831

**Published:** 2023-10-27

**Authors:** Zhixia Dong, Shan Wu, Jie Xia, Dongrui Liu, Yueqin Qian, Xinjian Wan

**Affiliations:** Digestive Endoscopic Center, Shanghai Sixth People’s Hospital Affiliated to Shanghai Jiaotong University School of Medicine, Shanghai, P. R. China

We herein report a unique case involving the coexistence of well-differentiated gastric-type adenocarcinoma and gastric adenocarcinoma of the fundic gland in a *Helicobacter pylori-* uninfected stomach.

An asymptomatic 51-year-old woman without *H. pylori* infection underwent a screening esophagogastroduodenoscopy at our hospital. The regular arrangement of collecting venules could be observed in the lower part of the stomach body and gastric angle under white-light endoscopy ( Fig. 1 a ), consistent with an *H. pylori* -uninfected mucosal background 1 2 .

**Fig. 1:**
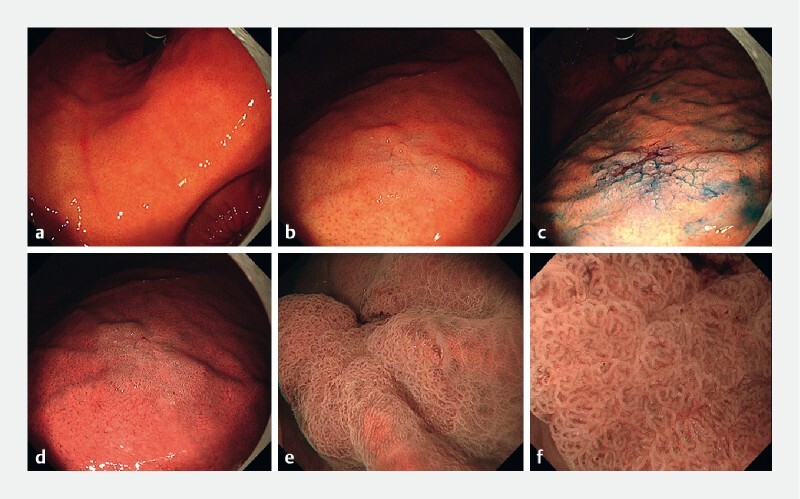
Endoscopic images showing: **a** no atrophy or intestinal metaplasia in the background gastric mucosa, with a regular arrangement of collecting venules visible in the lower part of the stomach body and gastric angle; **b** a type 0-IIa lesion on the anterior wall of the upper gastric body; **c, d** a clear demarcation line on narrow-band imaging (NBI) and indigo carmine dyeing; **e, f** an irregular microsurface pattern with a demarcation line on magnifying endoscopy with NBI.

On the anterior wall of the upper gastric body, a 15-mm slightly elevated (0-IIa) and whitish lesion (lesion A) was identified. Both narrow-band imaging (NBI) and indigo carmine dyeing revealed the lesion to have a clear boundary. Further examination using underwater magnifying endoscopy with NBI (ME-NBI) revealed an irregular microsurface pattern with a demarcation line ( Fig. 1 b–f ; Video 1 ), and a diagnosis of cancer was made 3 . A second 5-mm submucosal tumor-like elevated lesion (lesion B) with a discolored mucosal surface and dilatation of microvessels was seen at the greater curvature. ME-NBI showed a regular microsurface pattern without a demarcation line ( Fig. 2 ). According to the magnifying endoscopy simple diagnostic algorithm for early gastric cancer (MESDA-G) 4 , the diagnosis was noncancerous; however, as the endoscopic features on white-light imaging still strongly suggested a neoplastic lesion, lesion B was also diagnostically resected when endoscopic submucosal dissection (ESD) was performed for the lesion A.

**Video 1** Two simultaneous gastric cancers are identified in a *Helicobacter pylori-* uninfected stomach.


**Fig. 2:**
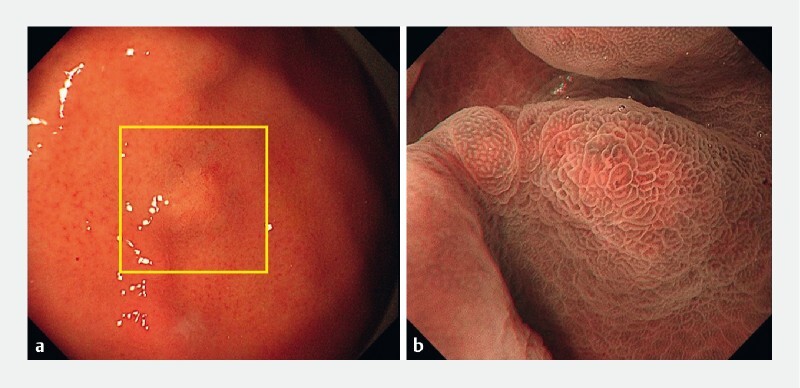
Endoscopic images of lesion B, which was seen at the greater curvature, showing: **a** a submucosal tumor-like elevated and discolored lesion with dilatation of microvessels on the surface; **b** a regular microsurface pattern without a demarcation line on underwater magnifying endoscopy with narrow-band imaging.

The final histologic examination showed that lesion A was a well-differentiated adenocarcinoma, which was confined to the mucosal layer without lymphatic or venous infiltration, and immunohistochemistry indicated the mucin genotype was gastric type. Lesion B was considered to be a gastric adenocarcinoma of the fundic gland (chief cell-predominant type) with a submucosal invasion depth of 800 μm, and negative vertical and horizontal margins.

The finding of simultaneous multiple gastric cancers in an *H. pylori-* uninfected stomach is extremely rare, so it is crucial that endoscopists are vigilant and pay more attention to minimize the risk of missed diagnosis.

Endoscopy_UCTN_Code_CCL_1AB_2AD_3AB

## Notes

Endoscopy E-Videos

https://eref.thieme.de/e-videos
*E-Videos* is an open access online section of the journal *Endoscopy* , reporting on interesting cases and new techniques in gastroenterological endoscopy. All papers include a high-quality video and are published with a Creative Commons CC-BY license. Endoscopy E-Videos qualify for HINARI discounts and waivers and eligibility is automatically checked during the submission process. We grant 100% waivers to articles whose corresponding authors are based in Group A countries and 50% waivers to those who are based in Group B countries as classified by Research4Life (see: https://www.research4life.org/access/eligibility/ ). This section has its own submission website at https://mc.manuscriptcentral.com/e-videos
